# A Singular Variety of the Disease Termed Chorea Sancti Viti

**Published:** 1799-03

**Authors:** G. C. Delarive

**Affiliations:** Assistant Physician to the Public Dispensary, London


### *A ſingular Variety of the Diſeaſe termed Chorea Sancti Viti: Communicated by* G. C. DELARIVE, *M. D. Aſſiſtant Phyſician to the Public Diſpenſary, London.*

*To the Editor of the Medical and Phyſical Journal.*

HAVING now under my care a caſe of the chorea, attended with ſome remarkable circumſtances, which may perhaps throw freſh light on the nature of that diſeaſe, I take the liberty to ſend you the particulars of it; anxious as I am to co-operate with you, as far as I am able, in an undertaking which cannot fail to prove both acceptable and uſeful to all medical men.— The caſe I allude to is the following:

Richard Hodge, aged fourteen years, a ſhoe-maker, was admitted into the Diſpenſary in Carey-ſtreet, on the 7th of January. So long as he remained in an erect, or horizontal poſition of body, he was perfectly well. While ſtanding, he was able to perform any voluntary motion whatever; as to extend his arm, and reach at any thing: he could alſo walk without any lameneſs, or dragging of one of his feet; ſo that he was to all appearance in perfectly good health. But on his attempting to ſit down, his legs and thighs inſtantly became agitated with the moſt violent convulſions; the right foot was firſt affected; it was alternately outwards and inwards. Theſe motions increaſed by degrees; at firſt he fell to kicking forward and backward, and afterwards to ſtamping the ground, which he did with a prodigious force. The ſame agitation took place, but in a leſs violent degree, and rather later, in the left leg and thigh. Theſe convulſive motions were not attended with any pain, unleſs that ariſing from the force with which he ſtruck the ground with his feet. By long ſitting down, inſtead of abating, they increaſed to ſuch a degree that, as he declared himſelf, he would have been knocked out of his chair, had he not ariſen and put himſelf in a ſtanding poſture, in which he found himſelf perfectly at eaſe.

Though he could at any time raiſe himſelf from his ſeat, and by ſo doing put an end to theſe violent contortions, while ſitting he had very little power over his legs; he was able, indeed, to ſtretch out one of them, but he could not retain it in that poſture above one or two ſeconds; nor could he, though ever ſo willing, check theſe convulſive motions in the ſmalleſt degree. The trunk of his body, his face, and his arms, were not, however, in the leaſt affected. When laid down on his bed, he was perfectly well; when made to ſit on a chair, with his legs placed in an horizontal poſition, they became convulſed, but apparently in a leſs degree than when his feet reached the ground. While ſtanding he could keep one of his legs and thighs bent againſt his body for ſome time, without any convulſions taking place; and when laid down in his bed, he could draw up his legs, and remain in that ſituation without any inconvenience. He was very diſtinct in his ſpeech, his mind did not appear in the leaſt affected, and all his natural functions were regular. He complained bitterly of his ſituation, not as being accompanied with any pain, but with conſiderable fatigue; as he was obliged to be on his legs the whole day, and when weary to lie down on the floor.

About nine weeks prior to his admiſſion to the Diſpenſary, he had been ſeized with giddineſs, ſickneſs, and a general ſhaking of his body; his arms and legs were agitated with ſlight convulſive motions, which always became worſe on ſitting down. He took bark without any benefit, but was relieved, for the ſpace of about three days, by the uſe of ſweating-powders. He was directed to be electrified twice a day, and to take a great deal of exerciſe. Under this regimen, which he continued for about one month, he recovered ſo far as to find himſelf pretty well: at laſt, he left off electrifying, and returned to his work; a few days after he found himſelf quite unable to ſit down for any length of time; and had been in the ſituation above deſcribed, a fortnight before he applied to the Diſpenſary.

On the 7th of January he was directed to take the *pil. cœrul.* j* three times a day;

* According to the New Nomenclature, called *pilulæ cupri*, each of which contains ſomewhat more than half a grain of the *cuprum ammoniacum* of the Edinb. Pharmacopœia.

a day; on the 9th he was precisely in the same state as before, the pills producing some degree of sickness on his stomach; he continued, however, to take them, and was also directed to have a blister applied on the os sacrum. The blister rose partially, and was removed on the next day, the 10th; yet he could sit down on this day for half an hour together, without feeling any inconvenience, and on the 11th, he was able to remain in that situation for an hour or two. The blistered place was healing very fast, when, on the 12th at night, he felt some slight return of his complaint. In consequence of this he was directed to keep the blister open, by dressing it with the ung. canth. he did so, and since that time has entirely recovered, and is now perfectly well. He is able now, (January 25, 1799) to sit down for a whole morning; the blistered place which is dressed with common cerate, is not yet healed up; he has never omitted regularly taking his pills. G. C. Delarive.

copoeia. We are informed by Dr. Du Bois, in the first Number, Vol. IV. of the Medical and Chirurgical Journal, edited by Prof. Hufeland, that the exhibition of this substance has been attended with the most beneficial effects, in an inveterate case of epilepsy. A girl, 16 years of age, who had been troubled with epileptic fits for three years, was directed to take *half a grain* daily of the cuprum ammoniacum; and after having produced nausea and vomiting, which were checked by the administration of the proper medicines, the dose was increased gradually to *five grains* daily. The patient completely recovered, having had no fits during seven weeks, whereas formerly she was regulary attacked twice and three times a week. W.

